# Books and Magazines

**Published:** 1900-04

**Authors:** 


### Books and Magazines.

ATLAS OF METHODS OF CLINICAL INVESTIGATION, with an Epitome of Clinical Diagnosis, and of Special Pathology and Treatment of Internal Disease. By Dr. Christfield Jakob, of Erlangen. Translated by Augustus A. Eshner, M. D., Professor of Clinical Medicine in the Philadelphia Polyclinic. W. B. Saunders, Philadelphia. Octavo, 182 colored illustrations on 68 plates and 64 illustrations in the text.

We know of no work equal to this in its special line, in the number, accuracy, and usefulness of its colored illustrations. The various appearances of the blood, the sputum, and the urine, in health and disease, are very beautifully as well as faithfully reproduced.

The series of illustrations representing the physical signs of disease of the chest and abdomen are of great practical value, and ought to be most helpful to the student. Moreover, the book is not simply an atlas. There are 259 pages of text, in addition to elaborate explanations of the illustration, and a thoroughly practical though brief account is given of clinical methods of investigation.

A TEXT-BOOK OF PATHOLOGY. By Alfred Stangel, M. D., Professor of Clinical Medicine in the University of Pennsylvania; Physician to the Philadelphia Hospital and to the Children's Hospital, Philadelphia. Octavo; 848 pages; 362 illustrations, many in colors. Second edition. Cloth, $4.00, net; half Morocco, $5.00, net.

This work is intended to present the subject of pathology in as practical a form as possible, and especially from the point of view of the "clinical pathologist." These objects have been faithfully carried out, and a valuable text-book is the result. An enormous amount of information is conveyed without any undue controversial matter being introduced, so that the reader is not confused with a multiplicity of views and opinions of numerous observers. It is a pleasure to find a book so plainly written.

It is most desirable that every practitioner should have an intelligent understanding of the cause of disease, the alterations in tissues caused by disease, and the way in which those changes take place. But a thorough and exhaustive knowledge of all these points would involve an amount of special study for which the general practitioner has neither the time nor the desire, and therefore a well-written text-book of moderate size, giving a reliable outline of the subject, must be welcomed by students and general practitioners. Such a work is the one before us.

---

A MANUAL OF MODERN SURGERY, General and Operative. By J. Chalmers Da Costa, M. D. 8vo., pp. 911, with 356 illustrations. Philadelphia: W. B. Saunders. Price, cloth, $4.00, net; half Morocco, $5.00, net.

Dr. Da Costa has elected to treat the common forms of surgical maladies in a direct, simple and concise way. He has admirably succeeded in putting before the student and the practitioner too, for that matter, a work which gives in a nutshell a view of modern surgical principles as applied directly to the treatment of disease.

The common fault of too great condensation giving false ideas of the subject, is avoided by omitting altogether the consideration of unusual diseases. The attention of the student is thus directed prominently toward those topics which he must most frequently consider in clinical work.

The reviewer is convinced from conversations with quiz-masters using this book that there is no better English work to be found for class-room instruction than this modest volume.

The new edition which is just issued is a great improvement on the old in every way, and in addition brings the consideration of every subject down to the present moment.

